# Postharvest Quality Improvement of Tomato (*Solanum lycopersicum* L.) Fruit Using a Nanomultilayer Coating Containing *Aloe vera*

**DOI:** 10.3390/foods13010083

**Published:** 2023-12-26

**Authors:** María L. Flores-López, Jorge M. Vieira, Cristina M. R. Rocha, José M. Lagarón, Miguel A. Cerqueira, Diana Jasso de Rodríguez, António A. Vicente

**Affiliations:** 1 Centre of Biological Engineering, Universidade do Minho, Campus de Gualtar, 4710-057 Braga, Portugal; lilianaflores@uadec.edu.mx (M.L.F.-L.); d8778@ceb.uminho.pt (J.M.V.); cmrocha@ceb.uminho.pt (C.M.R.R.); 2 Centro de Investigación e Innovación Científica y Tecnológica, Universidad Autónoma de Coahuila, Saltillo 25070, Coahuila, Mexico; 3 Novel Materials and Nanotechnology Group, Institute of Agrochemistry and Food Technology (IATA), Spanish Council for Scientific Research (CSIC), Calle Catedrático Agustín Escardino Benlloch 7, 46980 Paterna, Spain; lagaron@iata.csic.es; 4 International Iberian Nanotechnology Laboratory, Av. Mestre José Veiga s/n, 4715-330 Braga, Portugal; miguel.cerqueira@inl.int; 5 Plant Breeding Department, Universidad Autónoma Agraria Antonio Narro, Calzada Antonio Narro No. 1923, Colonia Buenavista, Saltillo 25315, Coahuila, Mexico; diana.jasso@uaaan.edu.mx

**Keywords:** nanomultilayer coatings, tomato, *Aloe vera* liquid fraction, gas barrier properties, postharvest quality

## Abstract

The effectiveness of an alginate/chitosan nanomultilayer coating without (NM) and with *Aloe vera* liquid fraction (NM+Av) was evaluated on the postharvest quality of tomato fruit at 20 °C and 85% relative humidity (RH) to simulate direct consumption. Both nanomultilayer coatings had comparable effects on firmness and pH values. However, the NM+Av coating significantly reduced weight loss (4.5 ± 0.2%) and molds and yeasts (3.5–4.0 log CFU g^−1^) compared to uncoated fruit (16.2 ± 1.2% and 8.0 ± 0.0 log CFU g^−1^, respectively). It notably lowered O_2_ consumption by 70% and a 52% decrease in CO_2_ production, inhibiting ethylene synthesis. Visual evaluation confirmed NM+Av’s efficacy in preserving the postharvest quality of tomato. The preservation of color, indicated by the Minolta color (a*/b*) values, demonstrated NM+Av’s ability to keep the light red stage compared to uncoated fruit. The favorable effects of NM+Av coating on enhancing postharvest quality indicates it as a potential alternative for large-scale tomato fruit preservation.

## 1. Introduction

Tomato (*Solanum lycopersicum* L.) is the second most important crop in the world after potatoes, with a production of approximately 11.6 billion tons of fresh weight in 2020 [1]. It is classified as a climacteric fruit, which means its ripening process is accompanied by an increase in respiration rate and ethylene production, leading to a relatively short postharvest shelf life [2]. For example, tomatoes stored at room temperature typically have a shelf life of around 7–11 d, depending on the variety [3,4]. The plant hormone ethylene is essential for normal fruit ripening, as it triggers various physical, physiological, and biochemical changes that enhance the appeal of tomatoes for consumption [5]. During the transition of tomato fruit from the mature green stage to fully ripe, which can occur on the plant or after harvesting, various quality parameters such as color, texture, and flavor undergo significant changes [6]. Additionally, the respiratory process brings about physiological consequences that are less desirable, including senescence, decay, chlorophyll degradation, and subsequent deterioration [7]. Transpiration, on the other hand, leads to shrinkage and weight loss of the produce by facilitating the movement of water vapor from the surface to the surrounding air [8].

Furthermore, tomatoes are susceptible to fungal attacks, such as *Rhizopus stolonifer* and *Penicillium expansum*, which can colonize injured fruits during harvesting and handling, rapidly spreading to adjacent fruits and causing significant losses [9]. Bacterial infections, including *Escherichia coli*, can also pose a risk to human health when contaminated tomatoes are consumed [10].

In general, it is crucial to control the processes of respiration and transpiration, as well as microbial contamination to extend the shelf life and maintain the quality of tomatoes. The conventional method used to delay and/or reduce ethylene production is storage at low temperatures, but this approach may cause chilling injury [11]. Pesticides and sanitizers are often employed to reduce pathogen levels; however, their application can result in residues that may exceed the maximum allowable limits, posing a serious problem for human health [12]. To address these challenges, recent research has focused on exploring improved and more efficient postharvest processing and preservation techniques. Among these techniques, the application of edible coatings has emerged as an alternative for extending the postharvest life of tomato fruit [2,13,14].

Edible coatings can modify the internal atmosphere of coated produce, creating a semi-permeable barrier against O_2_, CO_2_, and moisture. As a result, they can reduce respiration, water loss, and oxidation reactions [15]. Polysaccharide-based coatings are the most used materials to extend the shelf life of fruits and vegetables. Examples of these materials include sodium alginate [16], chitosan [17], galactomannans [18], pullulan [19,20], pectin [21], among others.

Recent studies have suggested that these coatings can exhibit improved functionality when used at nanoscale and applied in the form of a nanomultilayer on produce. Nanomultilayer coatings can be constructed by alternating the deposition of polyelectrolytes with opposite charges using the layer-by-layer (LbL) deposition technique [22]. Examples of this technique include chitosan/sodium alginate and chitosan/pectin coatings [15,23,24,25,26]. The objective is to combine their bioactivity and barrier properties, resulting in enhanced efficiency and an improved gas barrier compared to conventional coatings. Additionally, nanomultilayer coatings can serve as carriers for bioactive compounds, either directly or encapsulated, enabling controlled release of the active agents and prolonged bioactivity over time.

Recent studies have emphasized *Aloe vera*’s and its fractions’ potential in food preservation coatings. Tarangini et al. [27] combined *A. vera*’s bioactivity with sericin, chitosan, and glycerol, extending tomato shelf life by reducing deterioration for up to 21 d at 25 °C. *A. vera* gel-based edible coatings (60–80% gel) maintained higher levels of lycopene, ascorbic acid, sugar, carotenoids, flavonoids, and pectin, while reducing microbial counts on tomatoes stored at 10 °C for 30 d [28]. Additionally, *A. vera* liquid fraction displayed potent antioxidant and antifungal properties against key fungi during tomato postharvest [29,30].

However, there are no studies incorporating *A. vera* into nanomultilayer coatings. Therefore, the objective of this study was to assess the impact of incorporating *A. vera* liquid fraction into an alginate/chitosan nanomultilayer coating on the physicochemical parameters associated with the postharvest quality of tomatoes during storage at room temperatures (20 °C/85% RH, respectively). Additionally, the study aimed to investigate the role of the *A. vera* liquid fraction in controlling microbial spoilage and the respiration process of the tomato fruit.

## 2. Materials and Methods

### 2.1. Materials

Sodium alginate was obtained from Manutex RSX (Kelco International, Ltd., Portugal), and chitosan (91.23% deacetylation degree and high molecular weight) was purchased from Golden-Shell Biochemical Co., Ltd. (Taizhou, Zhejiang, China). Lactic acid of 90% purity and oxalic acid dehydrate were purchased from Merck (Darmstadt, Germany). Tween 80 was purchased from Acros Organics (Geel, Belgium). Sodium hydroxide was obtained from Riedel-de Haën (Seelze, Germany), and ascorbic acid was obtained from VWR (Radnor, PA, USA). Dichloran-rose Bengal-chloramphenicol (DRBC), glycerol, sodium chloride, and phenolphthalein were supplied by Panreac (Barcelona, Spain). The dye 2,6-dichlorophenol-indophenol (DCPIP) was obtained from Sigma (St. Louis, MO, USA). Plate count agar (PCA) and peptone bacteriological were purchased from HiMedia Laboratories (Mumbai, India).

The liquid fraction of *A. vera* (Aloe Vera Ecológico, Alicante, Spain) was obtained using the method described by Flores-López et al. [29].

Tomatoes (*Solanum lycopersicum* L.) of the round variety were selected at the turning-pink stage of ripening (average weight = 192 g), following the USDA standard tomato color classification chart (USDA, 1991), were purchased from a local supermarket in Braga, Portugal. The fruits were visually selected for uniformity in size, color, and absence of fungal infection, and were kept at 6 °C until use. Before the treatments were applied, the tomatoes were washed with a solution of sodium hypochlorite (0.05% *v*/*v*) for 3 min, then rinsing with distilled water, and air-dried at room temperature.

### 2.2. Experimental Design

The shelf-life analyses were performed at 20 °C and 85% RH (representing commercial storage conditions). Three different treatments were evaluated: uncoated tomatoes (control), nanomultilayer coating (NM), and nanomultilayer coating with *A. vera* liquid fraction (NM+Av), as presented in Table 1. The physicochemical and microbiological analyses were conducted at regular intervals (0, 3, 6, 9, 12, and 15 d) and respiration rate was evaluated daily for 8 d.

### 2.3. Coating Preparation

Polyelectrolyte solutions based on sodium alginate (Alg) and chitosan (Ch) were prepared according to the method described by Fabra et al. [22]. The concentrations of each polysaccharide, surfactant (Tween 80), and plasticizer (glycerol) were determined based on the spreading coefficient (Ws) studies on the tomato surface, as described by Casariego et al. [31]. Briefly, a 0.2% (*w*/*v*) Alg solution was prepared by dissolving Alg in distilled water and stirring at room temperature until complete dissolution. Glycerol (0.05%, *w*/*v*) and Tween 80 (0.05%, *w*/*v*) were added as a plasticizer and surfactant, respectively. The pH of the solution was adjusted to 7.0 using a 1 mol L^−1^ sodium hydroxide solution.

The Ch solution (0.6%, *w*/*v*) was dissolved in a solution of lactic acid (1.0%, *v*/*v*) and stirred until Ch was completely dissolved. Glycerol (0.1%, *w*/*v*) and Tween 80 (0.1%, *w*/*v*) were added, and the pH of the Ch solution was adjusted to 3.0 using a 1 mol L^−1^ lactic acid solution.

Subsequently, the *A. vera* liquid fraction was added to both the Ch (Ch-Av) and Alg (Alg-Av) coating solutions to achieve a final concentration of 0.6% or 0.2% (*w*/*v*), respectively, and mixed for 2 h at room temperature until homogenization. The concentrations of the *A. vera* liquid fraction were selected based on its reported range of bioactivity (antifungal and antioxidant) by Flores-López et al. [29], and considering the amounts of Alg and Ch used in the solutions (0.2% and 0.6% *w*/*v*, respectively), to obtain a polysaccharide: *A. vera* liquid fraction ratio of 1:1.

#### Zeta Potential

The zeta potential (ζ-potential) of each Alg and Ch coating solution was determined using a particle micro-electrophoresis instrument (Zetasizer Nano ZS-90, Malvern Instruments, Malvern, UK). Each sample was loaded into disposable capillary cells (DTS 1060, Malvern Instruments) and assessed at room temperature [2]. Additionally, the effect of adding *A. vera* liquid fraction to the coating solutions was evaluated in triplicate.

### 2.4. Nanomultilayer Coating Application on Tomato Fruit

The coatings were applied to the test groups using the LbL deposition technique, as shown in Table 1. No coating was applied to the control group (uncoated). Briefly, the tomatoes were immersed in a 0.2% (*w*/*v*) Alg solution at pH 7.0 for 10 s, and then rinsed with distilled water at the same pH (7.0). The samples were dried at 30 °C for 20 min in an oven with air circulation (Binder KBF, GmbH, Tuttlingen, Germany). The process was repeated using a Ch solution (0.6% *w*/*v*) at pH 3.0, followed by rinsing with distilled water at the same pH (3.0). This process was repeated with alternate deposition of a total of five layers (Alg-Ch-Alg-Ch-Alg). The immersion time, drying time between layers, and drying temperature were established based on preliminary tests. These conditions were selected to facilitate future scale-up of the process and application at the industrial level.

For each treatment, three replicates of five tomatoes (n = 15) were placed in trays and placed inside a controlled temperature and humidity chamber (Binder GmbH, Tuttlingen, Germany) at 20 °C and 85% RH. Temperature and RH during the shelf life and respiration tests were recorded using an iButton data logger (Thermochron, Dallas, TX, USA). Photographic documentation was utilized to capture the visual changes in the appearance of tomatoes by comparing the initial images of the treatments and the control over the storage period.

### 2.5. Physicochemical Analyses

#### 2.5.1. Weight Loss

The weight loss of five tomatoes per treatment was evaluated by weighing all samples using a precision balance (METTLER AE200, Mettler-Toledo, Giesen, Germany) at the beginning of storage (0 d) and during the experimental storage period. The percentage (%) of weight loss was determined using the following equation:

(1) $$Weight\;loss\left(\%\right)=\frac{W_{i}-W_{f}}{Wi}\times 100\;$$

where $W_{i}$ is the initial sample weight and $W_{f}$ is the final sample weight.

#### 2.5.2. Titratable Acidity (TA), pH, Soluble Solid Content (SSC)

At consistent intervals of 3 d during 15 d, three tomatoes from each treatment were analyzed. The samples were cut into small pieces, subsequently 50 g of each treatment was ground in a blender and filtered through Whatman filter paper no. 1 under vacuum. Titratable acidity (TA) was measured by utilizing 10 mL of previously obtained juice. Two drops of 1% (*w*/*v*) phenolphthalein were added, followed by titration using NaOH (0.1 mol L^−1^) using the 942.15 AOAC method. The results were expressed as a percentage (%) of citric acid. The pH of each treatment was determined using a pH meter (Hanna Instruments Inc., Bucharest, Romania) by directly submerging the electrode into the homogenized sample.

The juice from both the treatments and control groups was used to determine the soluble solid content (SSC) following the 932.12 AOAC method [32]. Briefly, a drop of the tomato juice was applied to a refractometer’s surface (HI 96801, Hanna Instruments Inc., Bucharest, Romania) calibrated with distilled water to measure the refractive index. Results were expressed as percentage (%). For all physicochemical tests, three samples per treatment were analyzed at each sampling time.

#### 2.5.3. Ascorbic Acid (AA) Determination

The ascorbic acid (AA) content was estimated using the DCPIP titration method of Ranggana [33] with some modifications. Briefly, the juice obtained from the fruit was centrifuged (Sigma 4K15, Sartorius, Göttingen, Germany) for 5 min at 12,000× *g* at room temperature. The supernatant (2.0 mL) was mixed with 5.0 mL of oxalic acid (4.0% *w*/*v*) and 2.0 mL of distilled water. The volume required to cause a color change in the DCPIP solution (24.0 g L^−1^ in distilled water) was recorded. A standard solution of ascorbic acid at a concentration of 20 g L^−1^ in distilled water was used as a reference. The results were expressed as mg kg^−1^ of AA per fresh weight (FW). All determinations were performed in triplicate.

### 2.6. Color

The color of the tomato skin was evaluated by measuring it with a Minolta colorimeter (CR 400; Minolta, Osaka, Japan). Average readings were taken at three points on the circumference of each fruit. The instrument was calibrated using a standard white color plate (Y = 93.5, x = 0.3114, y = 0.3190). The results were reported as Minolta color values according to the scale proposed by Batu [34] for tomato fruit, that indicates a direct correlation between the Minolta a*/b* ratio and the USDA ripening stages (Table 2), which were calculated using the following equation:

(2) $$Minolta\;color=\frac{a^{*}}{b^{*}}$$

where a* value corresponds to the degree of redness and the b* value represents yellowness in the Minolta colorimeter.

### 2.7. Firmness

Fruit firmness was determined using a texture analyzer (TA.XT, Stable Micro Systems, Godalming, UK). The tomato fruit was positioned at the center of the platform, and the force (*N*) required to penetrate 2.0 cm into the fruit was measured at the break point using a 6 mm flat-head stainless steel cylindrical probe. The test speed was set at 5.0 mm s^−1^. Firmness measurements were taken at the beginning and end of each test, and the results were reported as the mean ± SE (n = 10) and expressed in Newtons (*N*).

### 2.8. Microbiological Analyses

Microbiological analyses were conducted to count the total aerobic mesophilic microorganisms and molds and yeasts during the storage conditions, following the method described by Olivas et al. [35]. A sample weighing 10 g was aseptically collected from tomato surfaces and placed in a sterilized filter stomacher bag (VWR Scientific, West Chester, PA, USA) containing 90 mL of sterilized peptone water (0.1% *w*/*v*). The mixture was blended for 120 s using a Stomacher blender (3500, Seward Medical, London, UK). Serial decimal dilutions of the filtrate in 0.1% peptone water were pour-plated in duplicate on PCA agar and incubated at 37 °C for 2 d to count aerobic mesophilic microorganisms. Simultaneously, the same decimal dilutions were spread-plated on DRBC agar, a selective medium for the isolation and quantification of molds and yeasts. These plates were then incubated for 5 d at 25 °C.

The results were quantified and expressed as log colony-forming units per gram (log CFU g^−1^), encompassing the total count of aerobic mesophilic microorganisms as well as the combined count of molds and yeast observed on the selective DRBC agar plates. All analyses were performed with two replicates.

### 2.9. Gas Transfer Rate and Ethylene Production

The closed system method was used to measure the gas exchange (O_2_ and CO_2_) and ethylene (C_2_H_4_) production of tomato fruit. Acrylic air-tight cylindrical containers with a top lid fitted with a septum for gas sampling were used for each fruit and measured daily. A whole intact fruit sample was placed within each container, which was then placed in a controlled temperature and humidity chamber (Binder, Binder GmbH, Tuttlingen, Germany) to maintain the storage conditions. Evaluations were made daily for 8 d. Temperature and RH were recorded using an iButton data logger (Thermochron, Dallas, TX, USA) placed inside the container.

The O_2_ and CO_2_ contents were determined using a gas chromatograph (Bruker Scion 456, Markham, ON, Canada) equipped with two thermal conductivity detectors (TCD). The gas chromatograph had two columns: SS MolSieve 13 × (80/100), 2 m × 2 mm × 1/8′′ for O_2_ determination and BR Q PLOT, 30 m × 0.53 mm for CO_2_ measurement. Argon and helium were used as carrier gases, respectively. Calibration was performed using a mixture containing 10% CO_2_, 20% O2, and 70% N_2_. The C_2_H_4_ production was evaluated using a Varian Star 3400 CX (Palo Alto, CA, USA) gas chromatograph, coupled with a flame ionization detector (FID). The chromatograph was equipped with a vf-5 ms 30 m × 0.25 mm, 0.25 µm column. Helium, nitrogen, air, and hydrogen were used as carrier gases. Ethylene at 500 mg L^−1^ (Calgaz, Staffordshire, UK) was used as a standard for calibration.

The determination of gas transfer rate was performed with three replicates for each group of samples. Three full replicates were performed for both the control and the coated fruit groups. The O_2_ consumption and CO_2_ and C_2_H_4_ production rates were determined as described by Cerqueira et al. [36] with some modifications.

### 2.10. Statistical Analyses

The data analyses were conducted using FAUANL software v. 2015 [37] and Statistica software (release 7, edition 2004, Statsoft, Tulsa, OK, USA). Analysis of variance (ANOVA) was performed to determine significant differences. Mean values that were significantly different (*p* < 0.05) were separated using the Tukey test for a randomized block experimental design.

## 3. Results and Discussion

### 3.1. Physicochemical Analyses

#### 3.1.1. Weight Loss

**Figure 1:**
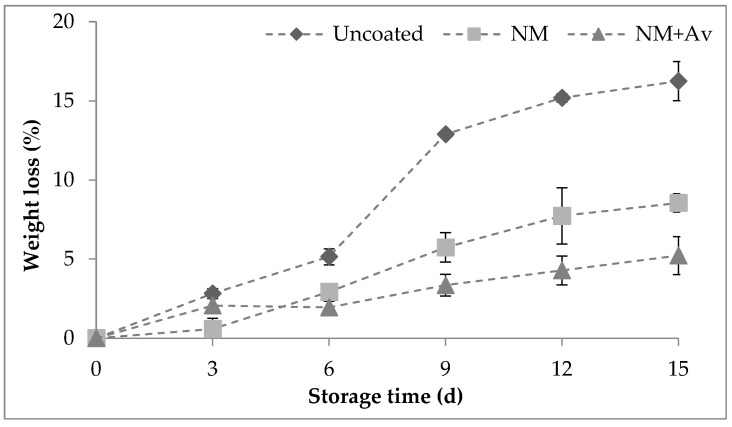
Tomato fruit weight loss (%) during storage at 20 °C/85% RH for 15 d. Values are the mean ± SE.

A weight loss above 5% is considered a limiting factor for the postharvest life of fruit crops, as there is a known relationship between this parameter, temperature, and storage time [38]. Figure 1 presents the weight loss of tomato fruit during storage at 20 °C and 85% RH. There was a significant difference between tomatoes coated with NM+Av coating and those coated with NM coating or uncoated tomatoes. This difference was more pronounced (*p* < 0.05) on the 15th d, with weight loss values of 5.2 ± 1.2% for tomatoes coated with NM+Av, and 8.5 ± 0.6% and 16.2 ± 1.2% for those coated with NM coating and uncoated tomatoes, respectively. The improved water barrier provided by the nanomultilayer coatings compared to uncoated tomatoes can be attributed to the electrostatic interactions between the Alg and Ch layers. This was corroborated by the ζ-potential values of the Alg solution (−60.40 ± 4.20 mV), which were lower and carried an opposite charge compared to the Ch solution (65.40 ± 3.70 mV). These interactions increase the tortuosity of the system, thereby reducing the diffusion of molecules through the coating materials [15].

The observed reduction in moisture loss and the subsequent improvement of postharvest quality can be attributed to the *A. vera* functionalization, which may be comprehended through its interaction with the hydrophilic groups inherent in Ch. The incorporation of *A. vera* liquid fraction into the Alg solution resulted in a significant increase in the charge (−45.50 ± 3.30 mV), potentially associated with the partial neutralization of Alg’s carboxylic groups by positively charged components of *A. vera* (e.g., proteins). However, there were no significant differences observed in the Ch coating solution upon the incorporation of the *A. vera* liquid fraction (72.20 ± 4.50 mV). These conditions guarantee the occurrence of electrostatic interaction between these polysaccharides even when functionalized. *A. vera*, known for its bioactive compounds, likely forms hydrogen bonds or other molecular interactions with Ch’s hydrophilic sites. This interaction alters the Ch-water dynamics, possibly by forming a protective barrier or modifying the surface properties of the Ch-based coating. Consequently, this impedes water permeation or enhances water vapor resistance, subsequently diminishing the rate of moisture loss from the coated fruit [39]. A study conducted by Morad et al. [40] indicated that tomatoes coated with *A. vera* gel also created a physical barrier that reduced the transfer of moisture from the inside of the fruit to the outside, attributed to its hygroscopic nature, the presence of hydrophobic compounds, and a higher polysaccharide content. Vieira et al. [30], also observed a significant decrease in weight loss in a chitosan-based coating containing *A. vera* liquid fraction after being stored at 5 °C and 90% RH for 25 d. This highlights the efficacy of such interactions in preserving produce quality during storage under specified conditions.

#### 3.1.2. Titratable Acidity (TA), pH, Soluble Solid Content (SSC)

The pH, TA, and SSC values for tomatoes under storage at 20 °C and 85% RH are presented in Table 3. The SSC values in tomatoes ranged between 3.6 and 4.6%, which is consistent with the values reported by Zapata et al. [41] for tomato fruit. The SSC values remained stable throughout the 15-d storage period, showing no significant differences between treatments, or compared to day 0. This stability suggests a maintained level of sweetness in the tomatoes, which is consistent with the results reported by Javanmardi and Kubota [42] for tomatoes stored at temperatures ranging from 25 to 27 °C. The pH values remained relatively steady during storage, yet an increase was noted in coated tomatoes by day 15 compared to day 0. This pH rise in coated tomatoes suggests an ongoing maturation, possibly influencing taste development while also being protected against weight loss, as shown in Figure 1.

On the other hand, the acidity of tomatoes significantly contributes to their taste and is intricately linked to the maturation process. However, the acidity does not change linearly over time. Research indicates varying trends, such as decreasing malic acid and increasing citric acid until the turning stage, while contrasting studies show a gradual rise in malic acid throughout maturation [13]. These variations partially explain the results obtained, as the TA values remained stable for all treatments during storage, despite observed changes in other maturation-related parameters such as color. The balance between SSC and TA changes might play a crucial role in taste and quality maintenance during storage.

#### 3.1.3. Ascorbic Acid (AA)

**Figure 2:**
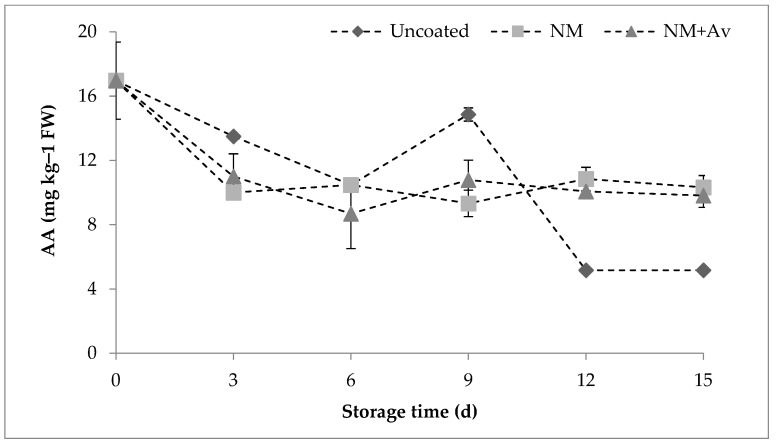
Ascorbic acid (AA) content (mg kg^−1^ FW) in tomato fruit during storage at 20 °C/85% RH for 15 d. Values are the mean ± SE.

In general, fruits are a natural source of AA, and its levels are reduced during maturation and processing. Due to its sensitivity, AA is used as an indicator of the severity of postharvest fruit damage. Figure 2 illustrates the concentration of AA in uncoated and coated tomatoes. A reduction in AA levels can be observed on the second day of analysis, which aligns with previous findings, and it is attributed to AA being used as a substrate or converted into sugars during ripening [43].

The AA levels were also observed to remain constant throughout the storage period in tomatoes coated with either of the coatings, with differences (*p* < 0.05) only being detected compared to uncoated tomatoes starting from day 12 (Figure 2). The significant reduction in AA content in uncoated tomatoes can be associated with the advanced ripeness of the fruit. This reduction may be attributed to the antioxidant function of AA, where ripening cells absorb higher levels of oxygen due to an increase in respiration rate, which is a characteristic physiological change in climacteric fruits and vegetables at ripeness [44]. The application of nanomultilayer coatings aided in reducing AA loss, although the incorporation of *A. vera* liquid fraction did not influence AA retention, as no significant differences were found between the two coatings. Similarly, multilayer systems such as the Ch-(β-cyclodextrin + trans-cinnamaldehyde complex)-pectin-based multilayer edible coating (with a thickness of 300 ± 1 µm) reported by Brasil et al. [45] demonstrated the ability to retain higher AA values in papaya compared to uncoated papaya during storage at 4 °C.

### 3.2. Color and Firmness

**Figure 3:**
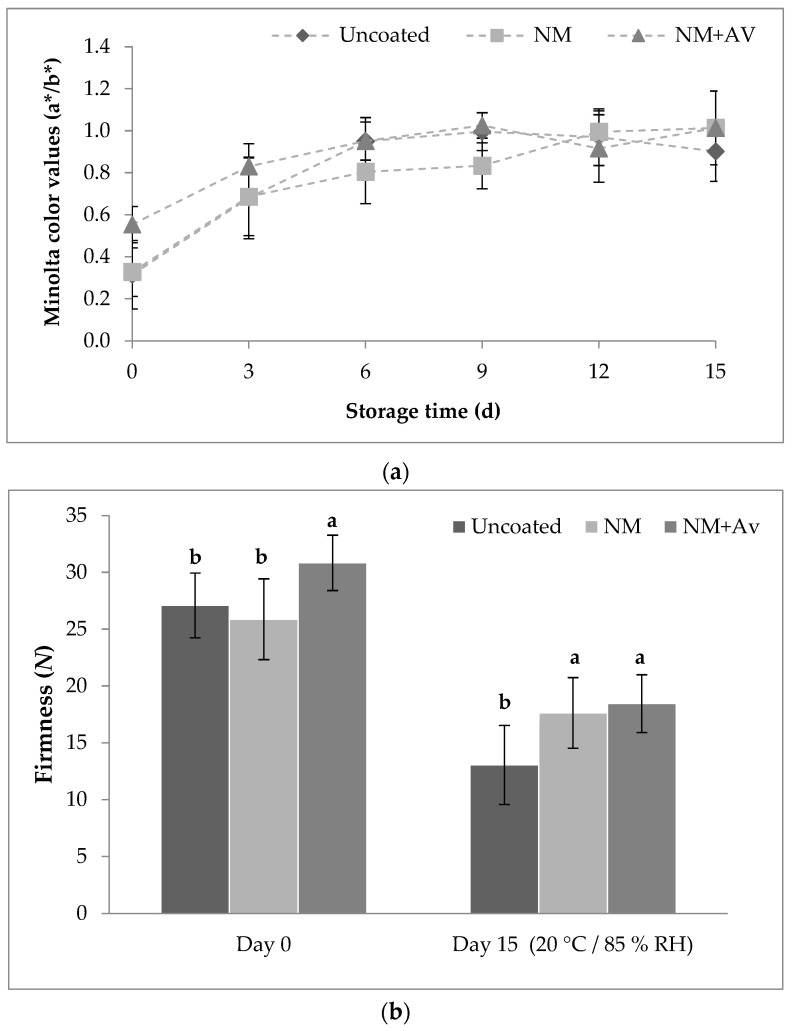
(**a**) Minolta color (a*/b*) values and (**b**) firmness of tomato fruit during storage at 20 °C/85% RH for 15 d. Values are the mean ± SE. For firmness, different letters on the same day indicate statistical differences (*p* < 0.05).

Color serves as an important indicator of ripeness and quality in tomatoes [6]. The a* value is used to monitor red color development and the degree of ripening in tomato fruit, while the b* value indicates yellow discoloration. Batu [34] provided a scale of a*/b* values to express the redness and its relationship with the maturation stage of tomatoes (Table 2). Figure 3a demonstrates that the color development, as indicated by an increase in Minolta color a*/b* values, was more pronounced (*p* < 0.05) in uncoated tomatoes throughout the storage period. However, the application of nanomultilayer coatings helped preserve the color attributes of the fruit, with the light red stage of tomatoes being sustained throughout the entire test. The effectiveness of *A. vera*-based coatings in reducing color development in table grapes and mushrooms has been previously reported by other researchers [46,47].

Regarding fruit firmness, the application of NM-Av at day 0 significantly increased the firmness of tomatoes compared to uncoated and NM-coated tomatoes. This increase may be attributed to the greater thickness of NM+Av (500 nm) compared to NM (420 nm) (Figure 3b). Similarly, Athmaselvi et al. [13] reported higher firmness in tomatoes coated with an *A. vera*-based coating. A significant decrease in firmness occurred at the end of the storage period, with uncoated tomatoes exhibiting the lowest firmness. The coated tomatoes maintained higher firmness, but no significant differences were found between the two types of nanomultilayer coatings studied. Consistent with these results, Ali et al. [6] reported lower loss of firmness in tomatoes coated with a gum arabic-based coating during storage at 20 °C and 80–90% RH. Fruit softening is a result of the degradation of cell structure and the internal composition of the cell wall by enzymes (e.g., hydrolases) acting on pectin and starch. These actions are closely linked to the progress of fruit ripening. The effect of coatings on delaying fruit softening is associated with their ability to act as a barrier for O_2_ uptake. The results of the gas transfer rate showed that coated tomatoes exhibited significantly lower O_2_ consumption than uncoated tomatoes, thereby slowing down metabolic activity and, consequently, the ripening process [48].

### 3.3. Microbial Analyses

**Figure 4:**
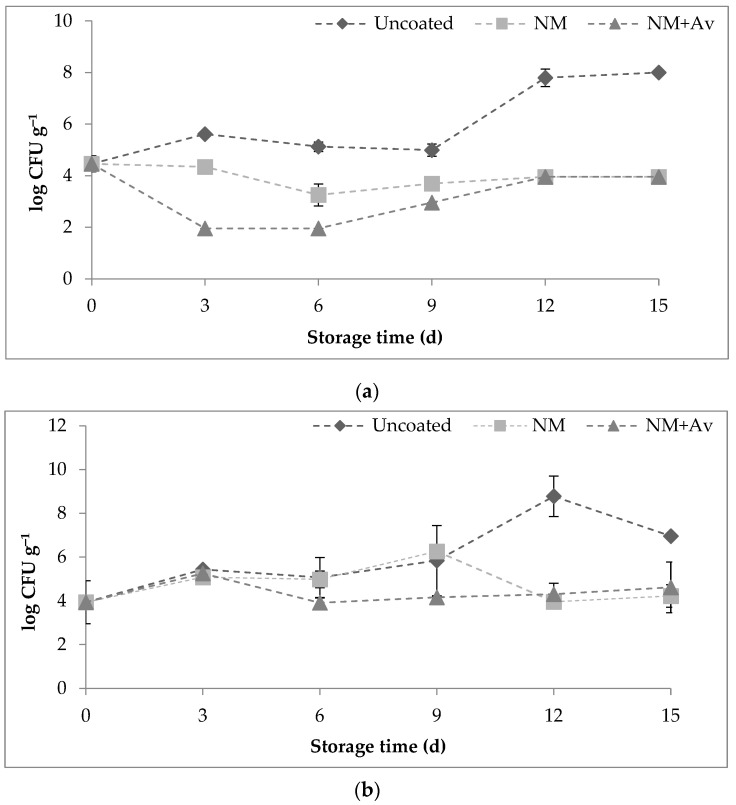
(**a**) Microbiological counting of molds and yeasts and (**b**) aerobic mesophilic microorganisms throughout storage time at 20 °C/85% RH. Values are the mean ± SE.

During the storage period, NM+Av exhibited better inhibition until day 9, and after day 12, no significant differences were found between tomatoes coated with both nanomultilayer coatings (Figure 4a). However, these treatments showed reduced (*p* < 0.05) populations of molds and yeasts (4.0 ± 0.0 log CFU g^−1^) compared to uncoated tomatoes (8.0 ± 0.0 log CFU g^−1^).

The antifungal activity of *A. vera* liquid fraction has been associated with the suppression of germination and inhibition of mycelial growth in fungi such as *Rhizoctonia solani*, *Fusarium oxysporum*, *Colletotrichum coccodes*, *B. cinerea*, and *P. expansum* [30,49]. These activities can be attributed to the presence of more than one active compound, although the specific mechanism of action is still unknown [47]. Recently, Vieira et al. [30] reported significantly lower counts of yeasts and molds on blueberry fruit coated with Ch- and Ch-liquid fraction of *A. vera*-based coatings after 25 d of storage at 5 °C and 90% RH. This activity was higher when *A. vera* was incorporated into the coating, although the authors also indicated a combination of the effects of Ch and *A. vera*.

The initial count of mesophilic microorganisms was 4.0 ± 0.7 log CFU g^−1^, and it increased during storage (Figure 4b). However, no significant differences in mesophilic counts were found between coated and uncoated tomatoes, but a slight reduction (*p* < 0.05) was observed at day 12 compared to uncoated samples.

**Figure 5:**
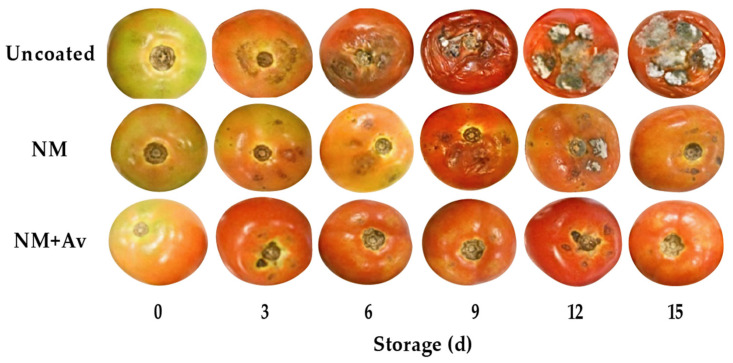
Tomato fruit’s gradual change throughout a 15-d storage period at 20 °C/85% RH.

The effectiveness of the evaluated nanomultilayer coatings was higher for yeasts and molds than for mesophilic microorganisms. Valverde et al. [47] also found the same behavior on table grapes coated with *A. vera* gel. It is supposed that the antimicrobial activity of *A. vera* cannot be sustained throughout storage, probably due to the stability of its bioactive compounds, mainly phenolic compounds and organic acids, which are responsible for its antimicrobial activity, as reported by Flores-López et al. [29]. Visual evaluation confirmed that the uncoated tomatoes had extensive spoilage on the surface after 15 d of storage (Figure 5).

### 3.4. Gas Transfer Rate and Ethylene Production

**Figure 6:**
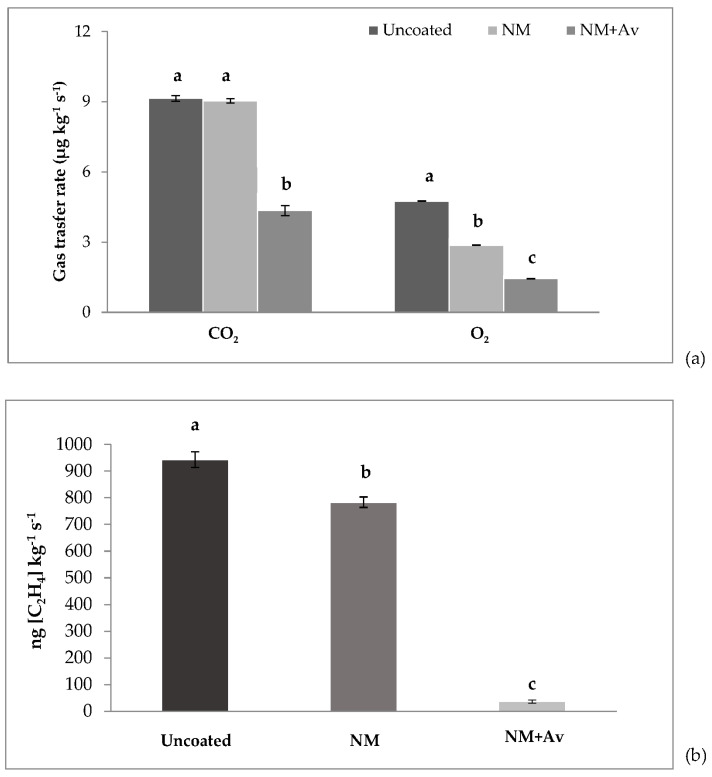
(**a**) CO_2_ and O_2_ transfer rates and (**b**) ethylene production during storage at 20 °C/85% RH for 8 d. Values are the mean ± SE. Different letters (a–c) indicate statistical differences between treatments (*p* < 0.05).

Innovation in the design of gas barrier materials plays a key role in the agro-food industry, as they can extend the shelf life of produce. Figure 6a shows the effect of coated treatments on CO_2_ production, in which a statistically significant reduction in the respiration rate was observed at day 8 for tomatoes coated with NM+Av compared to uncoated and NM-coated tomatoes.

NM+Av exhibited a lower gas transfer rate, with a 70% lower O_2_ consumption and 52% lower CO_2_ production after storage, compared to uncoated samples. Recent studies have demonstrated the effectiveness of κ-carrageenan and lysozyme nanomultilayer coatings in reducing O_2_ and CO_2_ exchange in fresh-cut and whole pears, as well as their role as a barrier to water loss [24]. Additionally, Medeiros et al. [23] associated the reduction in gas flow with the extension of shelf life in mangoes coated with a pectin and Ch-based nanomultilayer coating.

The ethylene production rate at the end of the experiment (day 8) showed differences between treatments (Figure 6b). These results indicate that the NM+Av coating did not significantly alter the gas balance in the tomato fruit, as it primarily reduced the respiration rates [50]. The observed reduction in ethylene production rates in tomatoes coated with NM+Av suggests influence on the fruit’s physiology. While the incorporation of *A. vera* into the nanomultilayer coating notably decreased ethylene production, it appears that this effect is not only attributable to altering gas balance. Instead, it is likely a combined outcome of the reduced respiration rates and enhanced gas barrier properties conferred by the NM+Av coating.

This reduction can be attributed to the fact that the application of the coating on the tomato’s surface restricts the permeation of respiratory gases. Additionally, the lower counts of yeasts and molds may influence the lower ethylene production in tomato fruit coated with NM+Av, suggesting that the accumulated ethylene in uncoated fruit is produced by fungi rather than the tomato fruit itself. The reduction in ethylene synthesis and the gas barrier properties of *A. vera* gel have been previously reported for climacteric fruits such as peaches, plums, nectarines [51], apples [52], and tomatoes [14].

Visual evaluation of the tomato fruit at the end of the storage conditions (20 °C/85% RH) also confirmed the significant effect of the NM+Av coating on maintaining quality and appearance (Figure 5). It provided a barrier against ethylene production and gas exchange between the inner and outer environments, resulting in a delay in the maturation process and an extension of the shelf life of the tomato fruit. Further work is required to scale up this technology to an industrial level to make its application feasible and accessible for producers.

## 4. Conclusions

The effectiveness of an alginate/chitosan nanomultilayer coating containing *A. vera* liquid fraction in extending the postharvest life of tomato fruit was evaluated. The application of nanomultilayer coatings (with and without *A. vera*) regulated maturation during storage conditions at 20 °C and 85% RH for 15 d, as it resulted in a reduction in the gas transfer rate in the coated tomato fruit. Among the coatings, NM+Av exhibited superior protective properties against weight loss, reduction in gas transfer rates, and ethylene production. Additionally, it effectively decreased microbial spoilage, thereby improving the overall quality of the tomato fruit. These beneficial properties of NM+Av make it a novel alternative for extending the postharvest quality of tomato fruit, which holds significant commercial value for both producers and consumers.

## Figures and Tables

**Table 1:** Treatments applied to tomato fruit.

Treatment	1st Layer	2nd Layer	3rd Layer	4th Layer	5th Layer
Uncoated					
Nanomultilayer coating (NM)	Alg	Ch	Alg	Ch	Alg
Nanomultilayer coating + *A. vera* liquid fraction (NM+Av)	Alg/Av *	Ch/Av **	Alg/Av *	Ch/Av **	Alg/Av *

Liquid fraction of *A. vera* (Av) at concentration of 0.2% (*w*/*v*) for alginate (Alg) * and 0.6% (*w*/*v*) for chitosan (Ch) **.

**Table 2:** Classification of USDA mature stages of tomato fruit according to Minolta color values.

Minolta Color Values (a*/b*)	USDA Tomato Color Stages
−0.59 to −0.47	Green
−0.47 to −0.27	Breaker
−0.27 to 0.08	Turning
0.08 to 0.60	Pink
0.60 to 0.95	Light red
0.96 to 1.21	Red

Adapted from Batu [34].

**Table 3:** Physicochemical properties of tomato fruit during storage at 20 °C/85% RH for 15 d.

Storage Time (d)		0	3	6	9	12	15
Uncoated	TA	0.3 ± 0.0 ^Aa^	0.2 ± 0.0 ^Ba^	0.2 ± 0.1 ^Aa^	0.3 ± 0.0 ^Aa^	0.2 ± 0.1 ^Aa^	0.2 ± 0.0 ^Aa^
	pH	4.5 ± 0.1 ^Aa^	4.5 ± 0.2 ^Aa^	4.5 ± 0.2 ^Aa^	4.5 ± 0.2 ^Aa^	4.5 ± 0.1 ^Aa^	4.6 ± 0.1 ^Aa^
	SSC	3.9 ± 0.2 ^Aa^	4.3 ± 0.2 ^Aa^	4.1 ± 0.2 ^Aa^	4.0 ± 0.4 ^Aa^	4.0 ± 0.3 ^Aa^	3.6 ± 0.1 ^Aa^
NM	TA	0.3 ± 0.0 ^Aa^	0.2 ± 0.0 ^Aa^	0.2 ± 0.0 ^Ba^	0.2 ± 0.0 ^Bb^	0.2 ± 0.0 ^Ba^	0.2 ± 0.0 ^Ba^
	pH	4.5 ± 0.1 ^Aa^	4.5 ± 0.2 ^Aa^	4.6 ± 0.1 ^Aa^	4.7 ± 0.1 ^Aa^	4.6 ± 0.0 ^Aa^	4.8 ± 0.0 ^Bb^
	SSC	3.9 ± 0.2 ^Aa^	4.6 ± 0.0 ^Ba^	3.9 ± 0.4 ^Aa^	3.9 ± 0.1 ^Aa^	4.0 ± 0.2 ^Aa^	3.8 ± 0.1 ^Aa^
NM+Av	TA	0.3 ± 0.0 ^Aa^	0.3 ± 0.0 ^Aa^	0.3 ± 0.1 ^Aa^	0.2 ± 0.0 ^Bb^	0.2 ± 0.0 ^Ba^	0.2 ± 0.1 ^Aa^
	pH	4.5 ± 0.1 ^Aa^	4.5 ± 0.2 ^Aa^	4.4 ± 0.2 ^Aa^	4.6 ± 0.2 ^Aa^	4.7 ± 0.1 ^Aa^	4.9 ± 0.1 ^Bb^
	SSC	3.9 ± 0.2 ^Aa^	4.0 ± 0.3 ^Aa^	4.0 ± 0.3 ^Aa^	4.0 ± 0.2 ^Aa^	3.9 ± 0.1 ^Aa^	4.5 ± 0.1 ^Ab^

TA = titratable acidity (% citric acid); SSC = soluble solid content (%). Means followed by the same lowercase letters in the columns and uppercase letters in rows did not show a statistically significant difference by Tukey’s test (*p* < 0.05).

## Data Availability

Data is contained within the article.
